# The Impact of Bt Corn on Aflatoxin-Related Insurance Claims in the United States

**DOI:** 10.1038/s41598-020-66955-1

**Published:** 2020-06-22

**Authors:** Jina Yu, David A. Hennessy, Felicia Wu

**Affiliations:** 1 Applied Economics, Division of Business and Management, Beijing Normal University - Hong Kong Baptist University United International College, Kowloon Tong, Hong Kong; 2 Department of Agricultural, Food, and Resource Economics, Michigan State University, Michigan, USA; 3 Department of Food Science and Human Nutrition, Michigan State University, Michigan, USA

**Keywords:** Plant biotechnology, Socioeconomic scenarios

## Abstract

Previous field studies have reached no collective consensus on whether Bt corn, the most commonly planted transgenic crop worldwide, has significantly lower aflatoxin levels than non-Bt isolines. Aflatoxin, a mycotoxin contaminating corn and other commodities, causes liver cancer in humans and can pose severe economic losses to farmers. We found that from 2001–2016, a significant inverse correlation existed between Bt corn planting and aflatoxin-related insurance claims in the United States, when controlling for temperature and drought. Estimated benefits of aflatoxin reduction resulting from Bt corn planting are about $120 million to $167 million per year over 16 states on average. These results suggest that Bt corn use is an important strategy in reducing aflatoxin risk, with corresponding economic benefits. If the same principles hold true in other world regions, then Bt corn hybrids adapted to diverse agronomic regions may have a role in reducing aflatoxin in areas prone to high aflatoxin contamination, and where corn is a dietary staple.

## Introduction

Bt corn is one of the most commonly planted transgenic crops worldwide. First produced in the United States in 1996, Bt corn contains transgenes from the soil bacterium *Bacillus thuringiensis*, which enable it to produce crystal proteins toxic to certain insect pests. Aside from improvements in corn growers’ yields^1–3^, Bt corn planting has also resulted in 11% less insecticide use for Bt adopters compared to non-adopters from 1998 to 2011^4^. Bt corn adoption among US corn growers rose from 19% in 2001 to 82% in 2018, including stacked events, which contain genes to combat multiple insect pests and confer herbicide and drought tolerance^5^. Bt corn planting has resulted in area-wide suppression of the European corn borer, improving yields for both adopters and non-adopters, even for vegetable growers^6,7^. This study advances the literature by examining another potential benefit of Bt corn adoption: reduced aflatoxin contamination, which leads to fewer aflatoxin-related crop insurance claims in the US.

Aflatoxins are mycotoxins (fungal toxins) produced primarily by the fungi *Aspergillus flavus and A. parasiticus* that commonly infect food crops such as corn, peanuts, pistachios, and almonds. Aflatoxin causes liver cancer, immune system dysfunction, and growth impairment in humans and animals^8^. The International Agency for Research on Cancer classifies “naturally occurring mixes of aflatoxins” as a Group 1 carcinogen^9^. Thus, over 100 nations worldwide have set regulatory standards for maximum tolerable levels of aflatoxin in food^10^. Even so, Liu and Wu estimated that between 25,000 and 155,000 aflatoxin-related liver cancer cases occur worldwide every year^11^. Few, if any, of these aflatoxin-related cancer cases occur in the US because, among other reasons, the US Food and Drug Administration (FDA) has set meaningful action levels for allowable aflatoxin in human food and animal feed: 20 micrograms per kilogram of aflatoxin in human food (20 parts per billion, or ppb), and varying standards for livestock and poultry. Although this ensures a safer food supply in the US, corn growers experience economic loss through rejected or discounted lots for excessively high aflatoxin levels^12,13^

**Figure 1:**
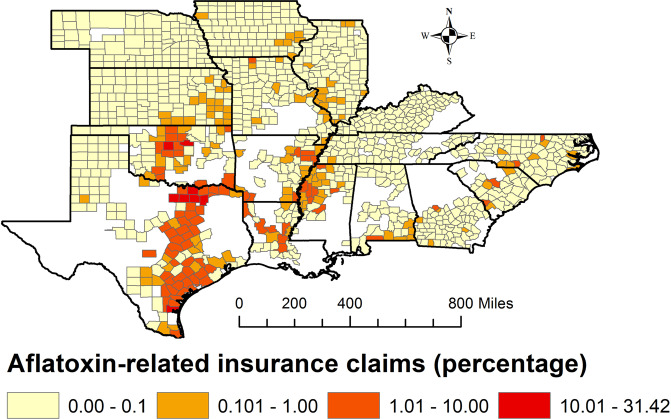
Number of aflatoxin-related insurance claims by county in 16 selected corn-planting states: 2001–2016. White portions in the sixteen states area were excluded from analysis because data on corn plantings were missing over some or all of the study period. (created in ArcMap 10.5.1, https://desktop.arcgis.com/en/arcmap).

Aflatoxin-related insurance claims in corn from 2001–2016 are shown in Fig. 1 (created in ArcMap 10.5.1, https://desktop.arcgis.com/en/arcmap), in 16 corn-planting states: Alabama, Arkansas, Georgia, Illinois, Iowa, Kansas, Kentucky, Louisiana, Missouri, Mississippi, North Carolina, Nebraska, Oklahoma, South Carolina, Tennessee, and Texas. These states were chosen based on completeness of data concerning Bt corn planting by county, aflatoxin-related insurance claims, and other factors necessary to conduct the analyses.

A secondary benefit to Bt corn planting, less well-known than insect control (its intended purpose), could be more important from public health and economic perspectives. In the late 1990s, it was first discovered that Bt corn had lower levels of the fungal disease Fusarium ear rot than non-Bt isolines, because it controls the insect pest European corn borer (*Ostrinia nubilalis*), which in turn reduces fungal infection by the endophytic fungus *Fusarium verticillioides*^14^. Hence, fumonisin – a mycotoxin produced by *F. verticillioides* that is associated with neural tube defects and growth impairment – is significantly lower in Bt corn than in non-Bt isolines^14–17^.

The results for fumonisin may suggest that any effort to reduce corn kernel damage would help to also reduce other fungal infections, including those contributing to aflatoxin contamination^18^. However, the relationship between Bt corn planting and aflatoxin concentrations has been less clear. In field trials, Bt corn has not consistently shown lower aflatoxin levels than non-Bt isolines^19–24^. One practical limitation of these past studies is that some were conducted in artificial field conditions: in many cases, the corn was inoculated with either insects conducive to *Aspergillus* infection (corn earworm, *Helicoverpa zea*; or fall armyworm, *Spodoptera frugiperda*) or the fungus itself. Also, the conditions that favor aflatoxin accumulation are complex. First, permissive conditions for *Aspergillus* infection are required, including high temperatures and drought stress^25^. Second, some corn hybrids are more or less easily affected by these environmental stressors^26^. Insect damage is important as well^27^. Bt corn, which controls for insect pest damage, would show an appreciable decrease in aflatoxin levels if the permissive conditions for aflatoxin accumulation were present and the insects that Bt corn was controlling were common in the area^28^.

What remains unknown is whether commercial Bt corn planting on US farms results in lower aflatoxin levels. This study sought to fill that gap in knowledge by determining if Bt corn planting results in fewer aflatoxin-related crop insurance claims by corn growers. Hence, the analysis would reflect what is actually happening in US commercial cornfields regarding the relationship between Bt corn planting and aflatoxin levels, and by doing so, would accommodate the variance described above because it is based on claims related to aflatoxin. We collected insurance claim data from the USDA Risk Management Agency (RMA), and Bt corn planting data from Kynetec, a survey company, from the period 2001–2016. The advantage of this approach is that it captures the reported economic losses to corn growers in a proxy manner, through the need of corn growers to make crop insurance claims for excessively high aflatoxin. This proxy measure is also useful because systematically collected quantitative data that directly measure aflatoxin levels are not available across the US. Any aflatoxin problems as captured in these crop insurance data are from actual pest, climate, and other environmental conditions in commercial cornfields in the US.

To assess the impact of both transgenic corn and climatic factors on aflatoxin problems in US corn, we developed a model that estimates causal relationships between the risk of aflatoxin-related insurance claims and Bt corn adoption rate, drought index, and climatic variables. Previous studies have shown that drought stress makes corn more susceptible to fungal infection^29–31^. Temperature is also critical for both maize growth and aflatoxin levels. Maize yields increase with temperatures between 12–25 °C, but decrease with temperatures above 30 °C^32^. In experiments, temperatures between 28–30 °C increase *A. flavus* growth, while temperatures above 37 °C decrease aflatoxin contamination^33,34^.

## Results

**Figure 2:**
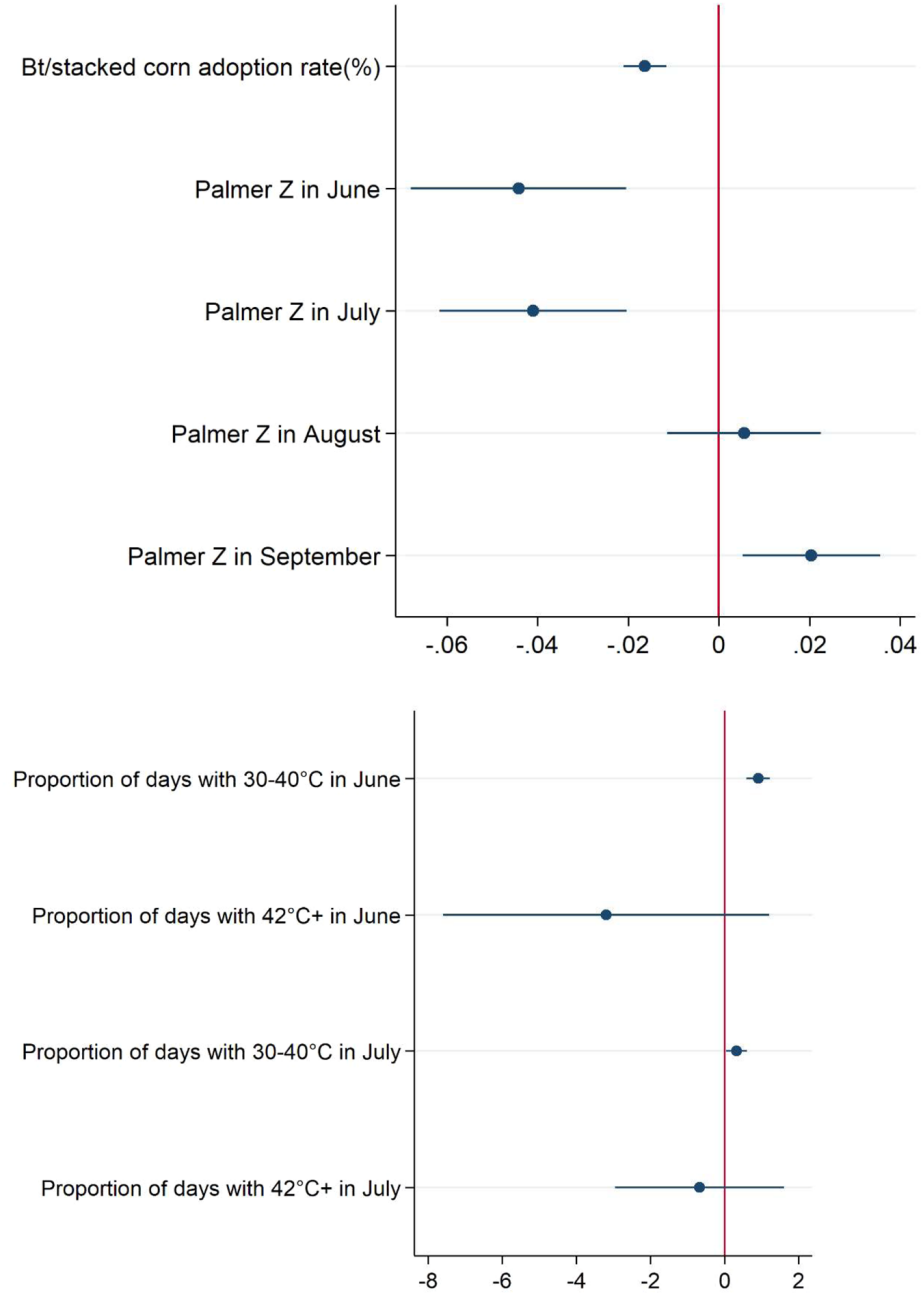
The marginal effect of Bt corn and climatic variables on risk of aflatoxin-related insurance claims. Bars represent 95% confidence intervals.

Table 1 provides three models: Model 1 shows the estimated marginal effect of Bt corn and climate variables on aflatoxin insurance claims (percentage). The marginal effects are the amount by which the expected value of a dependent variable (aflatoxin-related insurance claims) changes when a particular covariate increases by one unit (Bt corn planting) while other covariates are held fixed. Model 1 is also visualized in Fig. 2. Model 2 shows the estimated Viptera (Bt corn that specifically controls the pests corn earworm and fall armyworm, which have been associated with aflatoxin contamination^28^) and non-Viptera Bt effects. Model 3 indicates the estimated economic effects of each variable on aflatoxin insurance claim indemnity payouts. All models apply tobit models, which account for non-incidence of claims for most counties in most years. Since the indemnity payout depends on average insurance coverage level and insured area, these variables are controlled as well.

**Table 1:** Estimated marginal impacts of Bt corn, humidity/drought, and temperature on aflatoxin-related insurance claims and indemnities.

	Aflatoxin-related insurance claims (percentage)	Aflatoxin-related insurance claims (percentage)	$1,000 indemnities
Variables	(1)	(2)	(3)
Bt/stacked corn adoption rate (%)^b^	−0.016***	—	−14.10***^a^
	(0.003)	—	(3.041)
Viptera corn adoption rate (%)	—	0.252	—
	—	(0.163)	—
Non-Viptera Bt corn adoption rate (%)	—	−0.292**	—
	—	(0.139)	—
Palmer Z index (index range −6.44 ~ 11.58)			
Palmer Z in June	−0.044***	−0.129	−41.42***
	(0.012)	(0.200)	(14.53)
Palmer Z in July	−0.041***	−0.997***	−44.32***
	(0.011)	(0.292)	(11.24)
Palmer Z in August	0.006	−0.229	6.525
	(0.008)	(0.211)	(7.479)
Palmer Z in September	0.020***	0.046	16.84**
	(0.008)	(0.194)	(6.710)
Temperature in June (proportion of the temperature range, 0–1 value)			
Range 30–40 °C	0.908***	5.761**	893.1***
	(0.194)	(2.658)	(215.2)
Above 42 °C	−3.188	25.032	−3,576
	(2.727)	(25.471)	(2,302)
Temperature in July (proportion of the temperature range, 0–1 value)			
Range 30–40 °C	0.325**	10.206***	289.9**
	(0.140)	(3.395)	(137.5)
Above 42 °C	−0.675	2.466	−602.9
	(1.210)	(21.173)	(1,081)
Insurance			
Insurance coverage (ratio with range 0 and 1)	—	—	2,047**
	—	—	(797.1)
Insured area (hectare)	—	—	0.031***
	—	—	(0.010)
State Effects Controlled	Yes	Yes	Yes
Year Effects Controlled	Yes	Yes	Yes
Observations	12,127	4,198	12,127

^a^The marginal effect of Bt/stacked corn adoption, −14.1, in the model (3) means that an increase of one percentage point in Bt adoption rate in a county is associated with lowering aflatoxin-related insurance indemnities by $14,100 in that county, on average. ^b^Bt corn adoption rate is summation of Viptera corn adoption rate and Non-Viptera Bt corn adoption rate. Bootstrapped standard errors in parentheses, ***p < 0.01, **p < 0.05, *p < 0.1.

Results for alternative temperature combinations as well as details on year and state effects are provided in Supplementary Table 1. The *marginal effect* for Bt corn adoption rate, −0.016, indicates that an increase of one percentage point in Bt adoption in a county reduces aflatoxin-related insurance claims by about 0.016% in that county on average (model 1 in Table 1). This indicates that aflatoxin levels high enough to induce crop insurance claims are significantly lower in counties with high Bt corn adoption rates. To ensure robustness of the result, we conducted the same analyses using other econometric models: probit and fractional probit models (Supplementary Table 2). The results support an inverse association between Bt corn planting and aflatoxin risk. The marginal effects for the Palmer Z index, are significant and negative in both June and July where lower, negative values for the Palmer Z index correspond to drought conditions. The responses indicate that drought during these corn-growing months has led to greater aflatoxin risk. The positive sign in September indicates that wetter post-silking conditions increase aflatoxin problems.

The marginal effect for the June and July temperature range of 30–40 °C are significant and positive: consistent with previous experimental results^33,34^, these warm temperatures are positively correlated with higher aflatoxin incidence. On the other hand, temperatures above 42 °C in June and July are not statistically significant, suggesting that unfavorably high temperature ranges may not exists in field settings.

In Model 2, we included Viptera adoption rates and non-Viptera Bt adoption rates to examine the potential effect of Viptera traits introduced in 2011 (Details of result are reported in Supplementary Table 3). The Viptera effect was not statistically significant, while non-Viptera Bt corn planting reduced aflatoxin-related insurance claims by 0.292%. However, it is difficult to interpret this result as implying that any Viptera effect was not significant, because Viptera adoption rates have been low, particularly in the early years of adoption. These rates are reported in Supplementary Table 4.

For the sixteen states assessed in this study, the indemnities per year due to aflatoxin was US $10.6 million in 2001–2016 on average (Supplementary Table 5 reports aflatoxin-related insurance claims by states). The average payout per indemnified area was $415 per hectare. In the Supplementary Information, we infer that a markup in the range of 1.43 to 2 is appropriate when transforming indemnities payouts to losses. Given the assumption that non-insured areas have the same loss probability as insured areas, then aflatoxin causes losses ranging from $17.5 million to $24.5 million per year on average.

We also estimated hypothetical indemnities when Bt adoption rates were zero across the United States. Model 3 in Table 1 shows the estimated impacts of Bt corn adoption, drought, temperatures, insured area, and insurance coverage on the cost of aflatoxin-related indemnities. Here, insurance coverage provides the fraction of historical average yield below which yield shortfalls are recompensed. A markup adjustment is necessary to account for the fact that indemnities only cover losses beyond a large deductible. The estimated economic benefit of Bt corn planting due to reduced aflatoxin is defined as the markup adjusted difference between predicted indemnities from the fitted model and the hypothetical indemnities under zero Bt adoption. The difference was estimated at $120 million to $167 million per year for the selected 16 states. This estimated loss averted by Bt corn adoption is solely through reducing aflatoxin-related insurance claims, and does not take into account any economic effects of Bt traits on yield improvement or pesticide reduction. According to the USDA, the value of corn production over the selected sixteen states in 2001–2016 was $27.4 billion per year on average. Supplementary Table 6 reports value of corn production and also estimated benefit of Bt adoption. Thus, the estimated benefit corresponds to 0.4–0.6% of the total value of corn production in the 16 states.

These results suggest that Bt corn planting can reduce aflatoxin in US corn to an extent that causes a significant economic benefit to corn growers. If similar agronomic principles hold true in other world regions, then Bt corn planting may also reduce aflatoxin-related health and economic risks. On balance, climate projections suggest that more corn areas across the United States and worldwide will be vulnerable to aflatoxin in coming decades^35,36^. In this light, the trait can be viewed as a means of mitigating increased aflatoxin risk due to climate change. However, Bt corn is by no means the only possible route through which aflatoxin can be mitigated. Other interventions to reduce aflatoxin risk can be applied at the preharvest level: good agricultural practices, breeding resistance to field conditions that increase risk of *Aspergillus* infection, or biocontrol. Postharvest interventions to reduce aflatoxin include good storage practices and appropriate drying^37^. More research is warranted on whether Bt corn planting is associated with lower aflatoxin levels elsewhere worldwide, and on alternative control methods appropriate for high-risk areas for aflatoxin contamination of corn.

## Methods

### Materials

Aflatoxin-related insurance claims data were collected from the USDA Risk Management Agency (RMA) (https://www.rma.usda.gov/data/sob.html, accessed 4–26–18). According to the RMA, over 85% of US corn areas are insured through contracts underwritten by the RMA. Although these data do not include uninsured areas, and low levels of aflatoxin are not indemnified, it is the only data source that includes nationwide incidence of mycotoxin concentrations in corn at economically problematic levels. We measure aflatoxin incidence as percentage of all insured corn area in a county where indemnified losses are ascribed to mycotoxins in the RMA database.

Because aflatoxin is the only mycotoxin regulated by action levels by the United States Food and Drug Administration, we assumed that for the specific states chosen, mycotoxin-related indemnities (labeled “Mycotoxins [Aflatoxin]”) were for aflatoxin problems. The insurance claims data were collected only over the main part of the corn growing season (June to October) to rule out the possibility that claims are made for corn in storage. We narrowed our focus to 16 states where aflatoxin was the predominant mycotoxin causing economic damage within the data window, 2001–2016. These states are Alabama, Arkansas, Georgia, Iowa, Illinois, Kansas, Kentucky, Louisiana, Missouri, Mississippi, North Carolina, Nebraska, Oklahoma, South Carolina, Tennessee, and Texas. Among all year-county pairs in sixteen states, we only used observations with a record of insured corn planting, which amounted to 14,429 observations. We excluded 19 county-year interactions out of 14,429 that did not have *any* insured corn area from 2001–2016: Prairie Co., AR, in 2005; Pendleton Co., KY in 2002; Franklin Co., KY in 2004 and 2005; Clinton Co., KY in 2003; Granville Co., NC, in 2001; Custer Co., OK, in 2003 and 2006; Comanche Co., OK in 2007; Ellis Co., OK in 2001–2004; Cotton Co., OK in 2003; Fentress Co., TN in 2002; Crosby Co., TX in 2001 and 2002; Potter Co., TX in 2012; Grainger Co., TX in 2015. Thus, we had 14,410 observations after excluding these cases.

Bt corn adoption rates (including stacked events) were used as our explanatory variable of primary interest. We obtained data on Bt corn planting rates at the crop reporting district level in these 16 states from Kynetec Ltd., a survey and market analysis firm that specializes in agricultural markets. Each state contains four to sixteen districts, and each district includes about nine counties. Supplementary Table 7 reports Bt corn adoption rates by crop reporting district, aggregated by every two years.

We also included daily maximum temperatures from June to July as an explanatory variable, to determine which temperatures were most conducive to aflatoxin accumulation. The temperature variables are calculated as a proportion of the number of days with favorable/unfavorable temperatures. We tested multiple models with different temperature ranges to find favorable and unfavorable temperature levels in a field. Specifically, we created six pairs of potentially favorable and unfavorable average daily (including nighttime) temperature ranges: 26–36 °C vs. 38 °C and above, 26–36 °C vs. 40 °C and above, 26–36 °C vs. 42 °C and above, 28–38 °C vs. 40 °C and above, 28–38 °C vs. 42 °C and above, and 30–40 °C vs. 42 °C and above. We used the mean of temperatures observed from multiple weather stations within a county as a county level temperature. There are, on average, five weather stations within a county. Daily maximum temperatures were obtained from the US National Oceanic and Atmospheric Administration (NOAA)^38^.

We also included Palmer Z drought indices for several months as explanatory variables. The Palmer Z drought indices for each of the months of June through September were included as potential predictors of aflatoxin incidence^39^. Drought can affect aflatoxin accumulation on corn in several ways, including increasing plant stress that may make it more vulnerable to fungal infection and create an environment in which insect pests are more likely to damage corn. Palmer Z indices measure soil moisture availability over a month, rather than recent precipitation. Thus, it is a measure of the stock of water available for plant growth. It is available at the climate district level, accounting for water retention capacity of typical soils in the district. Each climate district includes several counties. NOAA provides the climate district level Palmer Z index as a monthly drought index (https://www1.ncdc.noaa.gov/pub/data/cirs/climdiv, accessed 10-24-18). Index values of 3.5 or higher indicate that the area is extremely wet for the area, while values of −2.75 or lower indicate that the area is extremely dry for the area^39^. Summary statistics and data sources are in Supplementary Table 8.

Among the 14,410 available observations, temperature and Bt adoption data are missing for some. These cases were excluded from analysis: 377 observations (168 counties) with incomplete Bt adoption data, and 1,906 observations (330 counties) with incomplete temperature data. As a result, 12,127 observations remained. We assumed that whether temperature or Bt adoption was observed is not systematically correlated with aflatoxin incidence. This assumption is supported by the market context. Aflatoxin control is not a major motivation for adopting Bt traits in corn. Major motives include removing the need for insecticide materials and spraying costs as well as yield damage avoidance. The assumption allows for an estimation without explicitly modeling incidence of missing data. However, data missing is also caused by discontinuous corn planting behavior. To check if incidence of missing data is correlated with aflatoxin occurrence, we included a dummy variable that has value zero if a county has all sixteen year data (no missing data) and value one otherwise. The statistically insignificant coefficient for the ‘missing’ dummy means that incidence of ‘missing’ is uncorrelated with aflatoxin-related insurance claims.

### Estimation Methods

To examine the impact of Bt corn and climate factors on aflatoxin accumulation, we used an econometric model. The reduced form is as follows:

1 $$\begin{array}{c}{y}_{i,t}^{\ast }={\hat{y}}_{i,t}^{\ast }+{c}_{i}+{u}_{i,t};\\ {\hat{y}}_{i,t}^{\ast }\equiv {\beta }_{B}{B}_{i,t}+{\sum }_{m\in \{6,7,8,9\}}{\beta }_{Z}^{m}{Z}_{i,t}^{m}+{\sum }_{m\in \{6,7\}}({\beta }_{F}^{m}{F}_{i,t}^{m}+{\beta }_{U}^{m}{U}_{i,t}^{m})+{\beta }_{M}{M}_{i}+{\beta }_{T}T+{\beta }_{S}S;\end{array}$$

where ${y}_{i,t}^{\ast }$ is the aflatoxin occurrence rate in county *i* in year *t*; ${B}_{i,t}\in [0,100]$ is the year *t* adoption rate for Bt and stacked genes for the county’s crop reporting district; ${Z}_{i,t}^{m}$ is the month *m* Palmer Z index for the climate zone in which the county is located (where 6 = June, 7 = July, 8 = August, 9 = September), ${F}_{i,t}^{m}$ is favorable temperature (for aflatoxin production) in the relevant county, month and year; ${U}_{i,t}^{m}$ is unfavorable temperature in the relevant county, month and year; ${M}_{i}$ is a vector of data missing dummy variables; *T* is a vector of year dummy variables; *S* is a vector of state dummy variables; ${c}_{i}$ is a county-specific unobserved factor; and ${u}_{i,t}$ is a normally distributed error term. We refer to the set of 12,127 observations as $H$.

For many US counties in many years, the aflatoxin percentage value was zero, i.e., no aflatoxin-related crop insurance claims were made. Thus, the standard linear model is not suitable for these data. Instead, we used a type I Tobit model that takes account of the zero bound, i.e., “latent” variable ${y}_{i,t}^{\ast }$ is not always observed. Type I Tobit models assume that ${y}_{i,t}^{\ast }$ satisfies the classical linear model assumptions^40^, but ${y}_{i,t}$ (aflatoxin insurance claims) never falls below zero (as it could in a standard linear model). Specification of the Tobit model is as follows:

2 $${y}_{i,t}=\,\max (0,{y}_{i,t}^{\ast });$$

where ${y}_{i,t}^{\ast }$ is as given in (1).

County specific and time invariant unobserved factors such as topography and soil characteristic can affect the aflatoxin incidence rate. Since they were not observed, we allowed county-specific unobserved factors, ${c}_{i}$, to be correlated with explanatory variables. Such a model is often called the Correlated Random Effects (CRE) model, in which we assume that the mean values of explanatory variables explain possible correlations^41,42^:

3 $${c}_{i}=\psi +{\bar{{\boldsymbol{x}}}}_{i}{\boldsymbol{\xi }}+{e}_{i};$$

4 $${v}_{i,t}|{{\boldsymbol{x}}}_{i,t} \sim {\rm{Normal}}(0,{\sigma }_{v}^{2});$$

where $\psi$ is a constant, ${\bar{{\boldsymbol{x}}}}_{i}$ is the vector of mean values for the explanatory variable vector ${{\boldsymbol{x}}}_{i,t}$ across time, ${e}_{i}$ is an error term, and ${v}_{i,t}={e}_{i}+{u}_{i,t}$ is composite error. The coefficient vector ${\boldsymbol{\xi }}$ measures the effect of time-averaged ${{\boldsymbol{x}}}_{i,t}$ on the unobserved county-specific feature ${c}_{i}$.

To estimate the CRE Tobit model, pooled maximum likelihood estimation (MLE) allowing serial correlation was used. With indicator function given by 1(·), having the value one when a given condition is satisfied and the value zero otherwise, the density of ${y}_{i,t}$ given ${{\boldsymbol{x}}}_{i,t}$ is

5 $$\begin{array}{c}f({y}_{i,t}|{{\boldsymbol{x}}}_{i,t})={\{1-\Phi ({J}_{i,t}/{\sigma }_{v})\}}^{1({y}_{i,t}=0)}{\{{{\sigma }_{v}}^{-1}\phi [({y}_{i,t}-{J}_{i,t})/{\sigma }_{v}]\}}^{1({y}_{i,t} > 0)};\\ {J}_{i,t}\equiv {{\boldsymbol{x}}}_{i,t}{\boldsymbol{\beta }}+\psi +{\bar{{\boldsymbol{x}}}}_{i}{\boldsymbol{\xi }};\end{array}$$

where $\phi (\,\cdot \,)$ is the standard normal probability density function and Φ(·) is the standard normal cumulative density function. Then the log likelihood for Correlated Random Effects Tobit is

6 $$ {\mathcal L} (\,\cdot \,)={\sum }_{(i,t)\in H}\log \,f({y}_{i,t}|{{\boldsymbol{x}}}_{{\boldsymbol{i}}{\boldsymbol{,}}{\boldsymbol{t}}},{\bar{{\boldsymbol{x}}}}_{i};\,{\boldsymbol{\beta }},{\sigma }_{v}^{2});$$

where $H$ is the set of observations, and ${\boldsymbol{\beta }}=\{{\beta }_{B},{\beta }_{Z}^{6},{\beta }_{Z}^{7},{\beta }_{Z}^{8},{\beta }_{Z}^{9},{\beta }_{F}^{6},{\beta }_{F}^{7},{\beta }_{U}^{6},{\beta }_{U}^{7},{\beta }_{M},{\beta }_{T},{\beta }_{s}\}$. The maximum likelihood estimators then are given by the $K$ dimensional parameter set $\varUpsilon ={\boldsymbol{\beta }}\cup \{\psi ,\xi ,{\sigma }_{v}^{2}\}$. The maximum likelihood estimators are the parameter values that solve ${{\rm{\max }}}_{\varUpsilon \in {{\mathbb{R}}}^{K}} {\mathcal L}$(·). Note that we follow the notation and explanation from Wooldridge (2010)^40^.

If the “true” error term is correlated with Bt adoption, then some of the error term is subsumed as part of the estimated Bt adoption effect, an issue that is often referred to as an “endogeneity problem.” For example, variations in soil conditions and populations of certain insects might be correlated with both Bt seed choice and aflatoxin incidence. In this case, the estimated effect of Bt adoption on aflatoxin already includes the effect from soil conditions. The instrumental variable (IV) method is a reliable method to correct for this form of bias^40^. An IV is a variable that is correlated with Bt adoption conditional on the other covariates, but is uncorrelated with the error term in the aflatoxin equation given that equation’s covariates. We used two IVs: expected yield and seed cost per expected yield. Farmers make decisions about adoption rates of Bt corn to maximize profits. Demand for Bt corn should increase as expected yield increases to the extent that the Bt trait is a value protecting input. Corn seed markets are imperfectly competitive, with two major providers over the period. They are also geographically separated because seed genetics must suit the local climate and soils. Seed providers should be able to charge more where demand is higher. Our second instrument is seed cost per expected bushel yield. Expected yield is measured out of sample, namely by crop district mean yield over 1991–2000 as reported by the USDA’s National Agricultural Statistics Service.

Two common estimators using an IV are the two stage least squares (2SLS) method and the control function (CF) method^43^. Since the 2SLS method for nonlinear models does not result in consistent estimations^44^, we used the control function (CF) approach. Intuitively, the CF approach estimates the causal relationship by detaching the part that might cause endogeneity problems, such as unobserved populations of certain insects and soil quality, from the error term. See Wooldridge for details on the CF approach^40,44^. Supplementary Table 9 indicates first stage regression results.

Since asymptotic variances for the two-step estimators are difficult to derive, we used the bootstrap standard error method with 1,000 replications^40,45^. The fact that we collected data from only counties with insurance uptake may cause selection bias; uninsured areas are not considered. However, we believe that this bias is negligible, as only nineteen observations were excluded due to the absence of crop insurance for corn over 16 years. Year dummy variables are included to account for differences in the natural and economic environments, including commodity price levels, pertinent farm bill legislation and weather conditions affecting insect and fungal populations. State dummy variables are included to control for state dependent features such as topography, proximity to the ocean and associated weather effects, and fungal populations in the soil.

To ensure the robustness of the results, we use other econometric models: specifically probit and fractional probit models. For the fractional probit model, the dependent variable are normalized to occur in the [0 1] range. For the probit model, a discrete variable with value one if aflatoxin related-insurance claims are reported and value zero otherwise is used as the dependent variable. We apply the control function approach with two IVs and correlated random effects to both probit and fractional probit models. For technical specifics on the method see Wooldridge (2010)^40^.

To measure any Viptera effect, we used the same model with the main model. The differences are 1) the time window is 2011–2016; 2) the seed cost per expected yield and expected yield are used as IVs for Viptera, and the yield effect is used as an IV for non-Viptera Bt. Viptera is more likely to have an endogeneity problem than non-Viptera Bt because a farmer is likely to adopt the Viptera trait when aflatoxin damage is more likely. However the coefficient on the first stage residual from Viptera adoption was not statistically significant. Therefore any endogeneity in the Viptera adoption choice seems unlikely. The results and F-statistics of IVs are reported in Supplementary Table 3.

### Method for estimating benefit of Bt adoption related to aflatoxin reduction

We defined the benefit of Bt adoption as the difference between loss due to aflatoxin and hypothetical loss when the Bt adoption rate was zero. Since we estimated loss due to aflatoxin by multiplying the aflatoxin-related indemnities by a markup factor, the benefit can be estimated as the markup times the difference between predicted indemnities from the fitted model using historic data and hypothetical indemnities without Bt adoption. In the Supplementary Information, we infer that a markup in the range of 1.43 to 2 is appropriate when transforming indemnities payouts to losses. Specifically, the benefit of Bt adoption can be written as follows:

7 $${{\rm{markup}}}^{\ast }{\sum }_{(i,t)\in H}({\hat{L}}_{i,t}{|}_{{B}_{i,t}=0,{{\boldsymbol{x}}}_{i,t}}-{\hat{L}}_{i,t}{|}_{{B}_{i,t},{{\boldsymbol{x}}}_{i,t}});$$

where ${\hat{L}}_{i,t}{|}_{{B}_{i,t},{{\boldsymbol{x}}}_{i,t}}$ is the conditional expectation of aflatoxin related indemnities in county *i* in year *t*, ${{\boldsymbol{x}}}_{i,t}$ represents the set of explanatory variables other than Bt adoption, and ${B}_{i,t}$ is the Bt adoption rate. The hypothetical indemnities ${\hat{L}}_{i,t}{|}_{{B}_{i,t}=0,{{\boldsymbol{x}}}_{i,t}}$ is defined as $E[{\hat{L}}_{i,t}|{B}_{i,t}=0,{{\boldsymbol{x}}}_{i,t}]$. We obtained coefficients by regressing indemnities on Bt adoption, the Palmer Z drought index, temperatures, year effect, state effect, insured area, and insurance coverage with a Tobit specification. Since indemnities depend on disease occurrence, insured area and insurance coverage, the insured area and the insurance coverage variables were included in the regression with the same explanatory variables as in the main model. The reduced form is as follows:

8 $$\begin{array}{c}{L}_{i,t}^{\ast }\equiv {\beta }_{B}{B}_{i,t}+{\sum }_{m\in \{6,7,8,9\}}{\beta }_{Z}^{m}{Z}_{i,t}^{m}+{\sum }_{m\in \{6,7\}}({\beta }_{F}^{m}{F}_{i,t}^{m}+{\beta }_{U}^{m}{U}_{i,t}^{m})\\ \,+\,{\beta }_{M}{M}_{i}+{\beta }_{T}T+{\beta }_{S}S+{\beta }_{A}{A}_{i,t}+{\beta }_{I}{I}_{i,t}+{c}_{i}+{e}_{i,t};\\ {L}_{i,t}=\,\max \,[0,{L}_{i,t}^{\ast }];\end{array}$$

where ${L}_{i,t}$ represents indemnities, ${A}_{i,t}$ is insured area in county *i* in year *t*, and ${I}_{i,t}$ is average insurance coverage in county *i* in year *t*. The insured area and the insurance coverage data are from USDA RMA. As with the main model, we controlled for county-specific and time-invariant unobserved factors, ${c}_{i}$, using the CRE model. The endogeneity problem was also addressed for these models using the control function approach with the expected yield and seed cost per expected yield as instruments. The bootstrap standard errors with 1,000 replications are reported in model 2 of Table 1.

The number of indemnities, ${\hat{L}}_{i,t}{|}_{{B}_{i,t}=0,{{\boldsymbol{x}}}_{i,t}}$ is estimated as

9 $$E[{L}_{i,t}|{B}_{i,t}=0,{{\boldsymbol{x}}}_{i,t}]=E[\,\max \,[{L}_{i,t}^{\ast },0]|{B}_{i,t}=0,{{\boldsymbol{x}}}_{i,t}]=\Phi ({\boldsymbol{x}}\hat{{\boldsymbol{\beta }}}/\sigma ){\boldsymbol{x}}\hat{{\boldsymbol{\beta }}}+\sigma \phi ({\boldsymbol{x}}\hat{{\boldsymbol{\beta }}}/\sigma );$$

where $${\boldsymbol{x}}\hat{{\boldsymbol{\beta }}}$$ denotes

10 $$\begin{array}{c}{\hat{\beta }}_{B}{B}_{i,t}+{\sum }_{m\in \{6,7,8,9\}}{\hat{\beta }}_{Z}^{m}{Z}_{i,t}^{m}+{\sum }_{m\in \{6,7\}}({\hat{\beta }}_{F}^{m}{F}_{i,t}^{m}+{\hat{\beta }}_{U}^{m}{U}_{i,t}^{m})+{\hat{\beta }}_{M}{M}_{i}+{\hat{\beta }}_{T}T+{\hat{\beta }}_{s}S\\ \,=\,0+{\hat{\beta }}_{A}{A}_{i,t}+{\hat{\beta }}_{I}{I}_{i,t}+{\hat{\beta }}_{c}{\tilde{c}}_{i};\end{array}$$

and $${\tilde{c}}_{i}=\psi +{\bar{{\boldsymbol{x}}}}_{i}{\boldsymbol{\xi }}$$.

Note that where ${y}^{\ast }$ is normally distributed then $y=\,\max \,[0,{y}^{\ast }]$ is truncated normal;

11 $$\begin{array}{c}{\rm{E}}(y|{\boldsymbol{x}})=\Pr (y=0|{\boldsymbol{x}})\times 0+\Pr (y > 0|{\boldsymbol{x}})E(y|{\boldsymbol{x}},y > 0)\\ \,=\,\Pr (u > -{\boldsymbol{x}}{\boldsymbol{\beta }}|{\boldsymbol{x}}){\rm{E}}(y|{\boldsymbol{x}},y > 0)=[1\,-\,\Phi (\,-\,{\boldsymbol{x}}{\boldsymbol{\beta }}/\sigma )]{\rm{E}}(y|{\boldsymbol{x}},y > 0)\\ \,=\,\Phi ({\boldsymbol{x}}{\boldsymbol{\beta }}/\sigma ){\rm{E}}(y|{\boldsymbol{x}},y > 0)=\Phi ({\boldsymbol{x}}{\boldsymbol{\beta }}/\sigma )\{{\boldsymbol{x}}{\boldsymbol{\beta }}+\sigma \phi ({\boldsymbol{x}}{\boldsymbol{\beta }}/\sigma )/\Phi ({\boldsymbol{x}}{\boldsymbol{\beta }}/\sigma )\}\\ \,=\,\Phi ({\boldsymbol{x}}{\boldsymbol{\beta }}/\sigma ){\boldsymbol{x}}{\boldsymbol{\beta }}+\sigma \phi ({\boldsymbol{x}}{\boldsymbol{\beta }}/\sigma ).\end{array}$$

The estimated benefits of Bt are reported in Supplementary Table 6.

## Supplementary information

Supplementary information.

Supplementary information 2.

## Data Availability

Insurance indemnity data used in this this study are available at http://www.rma.usda.gov/data, while weather data used are available at http://www1.ncdc.noaa.gov. Access to data from Kynetec was purchased. The company owns the data.
