# Author Correction: The Impact of Bt Corn on Aflatoxin-Related Insurance Claims in the United States

**DOI:** 10.1038/s41598-020-75643-z

**Published:** 2020-11-09

**Authors:** Jina Yu, David A. Hennessy, Felicia Wu

**Affiliations:** 1 Applied Economics, Division of Business and Management, Beijing Normal University - Hong Kong Baptist University United International College, Guangdong, China; 2 Department of Agricultural, Food, and Resource Economics, Michigan State University, East Lansing, Michigan USA; 3 Department of Food Science and Human Nutrition, Michigan State University, East Lansing, Michigan USA

Correction to: *Scientific Reports* 10.1038/s41598-020-66955-1, published online 22 June 2020

The original version of this Article contained errors.

Affiliation 1 was incorrectly given as ‘Applied Economics, Division of Business and Management, Beijing Normal University - Hong Kong Baptist University United International College, Kowloon Tong, Hong Kong’. The correct affiliation is listed below:

Applied Economics, Division of Business and Management, Beijing Normal University - Hong Kong Baptist University United International College, Guangdong, China

Additionally, the Abstract contained an error.

“Previous field studies have reached no collective consensus on whether Bt corn, the most commonly planted transgenic crop worldwide, has significantly lower aflatoxin levels than non-Bt isolines.”

now reads:

“Previous field studies have reached no collective consensus on whether Bt corn, one of the most commonly planted transgenic crops worldwide, has significantly lower aflatoxin levels than non-Bt isolines.”

These errors have now been corrected in the HTML and PDF versions of the Article.

