# Supplementary material for: The Impact of Bt Corn on Aflatoxin-Related Insurance Claims in the United States
Source: Sci Rep. 2020 Jun 22;10:10046. doi: 10.1038/s41598-020-66955-1 (PMC7308289; doi:10.1038/s41598-020-66955-1)
Supplement: Supplementary file 1 — Supplementary information. [file 41598_2020_66955_MOESM1_ESM.docx]

**Supplementary Information**

**The impact of Bt corn on aflatoxin-related insurance claims in the United States**

Jina Yu^1^, David A. Hennessy^2^, Felicia Wu^2,3*^

^1^Applied Economics, Division of Business and Management, Beijing Normal University - Hong Kong Baptist University United International College

^2^Department of Agricultural, Food, and Resource Economics, Michigan State University.

^3^Department of Food Science and Human Nutrition, Michigan State University.

Address correspondence to F. Wu ([fwu@msu.edu](mailto:fwu@msu.edu))

**This Supplementary Information contains:**

**Supplementary Text**

**Supplementary Tables 1-9**

Supplementary Text

Crop insurance in the United States

Crop insurance in the United States is a public-private partnership, whereby government actuaries set rates and insure much of the crop-related risk to farmers, while private firms market and retain some of the contracts. Public involvement commenced during the 1930s, and public support has expanded over the years. It is now the centerpiece of public support for incomes in crop agriculture. Details on the program’s history can be found in Glauber (2013)^1^. Public subsidies come in various forms, including rate-setting services, compensation for administration costs, and subsidized premiums. Since the late 1990s, the most widely used contracts for the main crops have been revenue insurance and yield insurance, whereby loss beyond a fraction of projected average revenue or yield is indemnified. Coverage levels offered range from 50% to 90% of projected revenue, depending on insurance contract chosen and location. The entity within the US Department of Agriculture that administers the program is called the Risk Management Agency, or RMA.

A critical feature of the process is loss assessment, for which procedures are described in a frequently updated Loss Adjustment Manual Standards Handbook: <https://www.rma.usda.gov/handbooks/25000/2018/18_25010-2h.pdf>. For most contracts, when a farmer makes a claim of a nature that requires a visit, an employee or contracting agent from the crop insurance marketing company that originated the contract visits the insured land tract to assess losses. The adjustor provides a report that includes, among other data, the primary cause of loss. Several months after the end of the crop growing season, reported data are aggregated to county level of analysis and summary reports are placed in the public domain, see <https://www.rma.usda.gov/data/cause.html>. Cause of loss data are available since 1948, but loss area and month of loss have only been available since 2001. Reported causes of crop loss include - among others - drought, excess moisture/precipitation/rain, freeze, heat, decline in price, plant disease, insects, wildlife, wind, and mycotoxins (aflatoxin).

Finding a favorable / unfavorable temperature range

We started by testing a variety of temperature ranges as favorable and unfavorable temperatures for aflatoxin accumulation, respectively. **Supplementary Table 1** shows the estimated coefficients for six pairs of potentially favorable and unfavorable temperature ranges. We excluded, among different bands of the favorable temperature ranges, those that did not show positive correlations with aflatoxin-related insurance claims. Since the temperature range 30-40°C has a positive coefficient and the range was only paired with ≥ 42°C, the range 30-40°C and ≥ 42°C (model 6) were chosen as favorable and unfavorable temperature ranges for aflatoxin accumulation, respectively.

Inferring total loss

We estimated the economic loss due to aflatoxin, as well as the economic benefit of Bt corn specifically through reducing aflatoxin. The economic loss from aflatoxin is calculated based on the amount of indemnity payout attributed to aflatoxin. Since indemnity payout is lower than total loss, we calculate mark up to transform indemnities payouts to losses. Suppose that the average corn crop insurance coverage level over the period is 70%, with a corn price of $3 per bushel. Average yield is assumed to be 400 bushels per hectare. In addition, we assume a uniformly distributed loss. Then revenue protected is $1,200 per hectare. Seventy percent coverage, which was typical for corn in most southern states during 2001-2016, would provide $840 total coverage per hectare. If revenue *L* is less than $840, then the indemnity is $840-*L*. Therefore, the indemnity is the maximum of 0 and 840-*L*. Upon integrating over the relevant domain, we obtain , which is somewhat lower than $415, the average indemnity per aflatoxin occurred area for 2001-2016 (**Supplementary Table 5**). An integration over the entire domain yields , suggesting a markup of when transforming indemnity payouts to losses. Alternatively, if it is assumed that losses are total, as in issuing a total destruction order, then the indemnity paid would be $840 per hectare, loss would be $1,200 per hectare, and the markup would be 1.43. A third plausible possibility is that all indemnity payments amount to $415 per hectare. If we solve , then the certain market revenue, conditional on the aflatoxin loss event occurring, that gives a $415 indemnity, is and total loss per event is $1,200-$425=$775 so that the markup is . Thus, we infer that a markup in the range of 1.43 to 2 is appropriate.

Calculation of marginal effect

The estimated marginal effect of Bt corn on aflatoxin is provided to capture the response of aflatoxin-related insurance claims upon a one percentage point increase in Bt adoption. The estimated marginal effect varies across the population because other model variables differ across the population. In order to capture a representative marginal effect we used the unconditional mean, i.e., the average marginal effect is calculated by $[N^{-1}\sum_{i=1}^{N} \Phi(x_{t}\hat{\beta}+\hat{\psi}+\bar{x}_{i}\hat{\xi}/\hat{\sigma}_{v}^{2})]\hat{\beta}_{j}$. This summary statistic is commonly called the Average Partial Effect (APE). When the model is non-linear, as with our model, then the APE is a more accurate estimation of the marginal effect.

Supporting References and Notes

1. Glauber, J.W. The growth of the federal crop insurance program, 1990–2011. *American Journal of Agricultural Economics* **95**, 482-488 (2013).

Supplementary Table 1. Estimated impact of Bt corn, humidity, and temperature on aflatoxin–related insurance claims using Tobit model (marginal effects and coefficients).

See Supplementary_Table.xlsx

Supplementary Table 2. Estimated marginal impact of Bt corn, humidity, and temperature on aflatoxin–related insurance claims using Probit and Fractional Probit models.

See Supplementary_Table.xlsx

Supplementary Table 3. Estimated marginal impact of Viptera and non-Viptera Bt corn on insurance claims.

See Supplementary_Table.xlsx

Supplementary Table 4. Viptera adoption rates (%) by states over two-year intervals, 2011-2016. ^a^

|  | 2011-2012 | 2013-2014 | 2015-2016 |
| --- | --- | --- | --- |
| AL | 0.5 | 5.4 | 10.3 |
| AR | 1.3 | 11.9 | 9.1 |
| GA | 0.0 | 12.0 | 10.2 |
| IL | 1.4 | 21.3 | 19.9 |
| IA | 1.4 | 16.1 | 21.9 |
| KS | 4.3 | 21.3 | 36.4 |
| KY | 1.9 | 24.9 | 30.1 |
| LA | 0.4 | 4.8 | 2.3 |
| MS | 1.8 | 4.6 | 17.1 |
| MO | 2.5 | 24.8 | 43.6 |
| NE | 2.8 | 21.9 | 31.9 |
| NC | 1.1 | 6.1 | 11.9 |
| OK | 0.0 | 19.1 | 30.5 |
| SC | 4.3 | 4.4 | 10.8 |
| TN | 3.6 | 18.1 | 25.4 |
| TX | 6.4 | 11.9 | 26.9 |
| Sixteen States Total | 2.2 | 18.8 | 25.8 |

^a^ Two years average value is calculated by mean value of each year's adoption rate

Supplementary Table 5. Summary of aflatoxin related insurance claims (indemnities and percentage), time averages 2001-2016 ^.a^

|  | (A)  Aflatoxin related  indemnities per year  (1,000 $) | (B)  Indemnities per insured area  (US $/hectare) | (C)  Indemnity per area reporting aflatoxin as primary cause of loss (US 1,000$/hectare) | (D)  Aflatoxin related claims (%) ^b^ |
| --- | --- | --- | --- | --- |
| AL | 11.4 | 0.16 | 285 | 0.06 |
| AR | 1,040 | 7.04 | 733 | 0.96 |
| GA | 15.5 | 0.15 | 588 | 0.03 |
| IL | 756 | 0.20 | 676 | 0.03 |
| IA | 638 | 0.13 | 449 | 0.03 |
| KS | 391 | 0.28 | 575 | 0.05 |
| KY | 14.4 | 0.04 | 854 | 0.00 |
| LA | 289 | 1.47 | 365 | 0.40 |
| MS | 2,490 | 10.51 | 894 | 1.18 |
| MO | 355 | 0.33 | 658 | 0.05 |
| NE | 32.7 | 0.01 | 681 | 0.00 |
| NC | 104 | 0.37 | 511 | 0.07 |
| OK | 255 | 2.70 | 443 | 0.61 |
| SC | 17.6 | 0.17 | 148 | 0.12 |
| TN | 49.0 | 0.23 | 696 | 0.03 |
| TX | 4,150 | 5.46 | 264 | 2.07 |
| Sixteen States Total | 10,613 | 0.63 | 415 | 0.15 |

^a^ Data for supplementary table 5 cover all counties in the set, including those not included in our regression due to unobserved covariates.

^b^ Aflatoxin-related claims are defined by 100 times the gross area lost due to aflatoxin divide by insured area.

Supplementary Table 6. Value of corn production and Tobit model estimates of Bt adoption benefits in sixteen states.

| Year | Value of corn production ^a^ | Loss due to aflatoxin ^b^ | Benefit of Bt adoption using aflatoxin indemnities | |
| --- | --- | --- | --- | --- |
|  | Million $ | Million $ | Million $ | Estimated benefit of Bt over Value of Corn production (%) |
| 2001 | 11,600 | 0.79-1.1 | 0.64-0.9 | 0.01-0.01 |
| 2002 | 13,020 | 15.6-21.8 | 23.9-33.5 | 0.18-0.26 |
| 2003 | 14,993 | 8.02-11.2 | 8-11.2 | 0.05-0.07 |
| 2004 | 15,256 | 0.1-0.14 | 4.5-6.3 | 0.03-0.04 |
| 2005 | 13,402 | 35.4-49.6 | 89.6-125.3 | 0.67-0.94 |
| 2006 | 19,560 | 10.3-14.4 | 64.6-90.4 | 0.33-0.46 |
| 2007 | 34,665 | 5.47-7.65 | 77-107.7 | 0.22-0.31 |
| 2008 | 30,318 | 31.2-43.7 | 140.1-195.9 | 0.46-0.65 |
| 2009 | 28,689 | 29.6-41.5 | 140.2-196.1 | 0.49-0.68 |
| 2010 | 38,707 | 49.0-68.6 | 236.2-330.4 | 0.61-0.85 |
| 2011 | 46,412 | 31.2-43.7 | 264.9-370.4 | 0.57-0.8 |
| 2012 | 41,762 | 54.3-76.0 | 457.3-639.6 | 1.1-1.53 |
| 2013 | 36,515 | 7.09-9.92 | 167.2-233.8 | 0.46-0.64 |
| 2014 | 32,236 | 0.8-1.12 | 81.1-113.4 | 0.25-0.35 |
| 2015 | 29,952 | 0.14-0.2 | 72.8-101.8 | 0.24-0.34 |
| 2016 | 31,540 | 0.69-0.96 | 83.3-116.4 | 0.26-0.37 |
| Average per year | 27,414 | 17.5-24.5 | 119.5-167.1 | 0.44-0.61 |

^a^ Value of corn production data over the sixteen states per year come from the USDA National Agricultural Statistics Service (NASS) ([https://quickstats.nass.usda.gov/](https://urldefense.proofpoint.com/v2/url?u=https-3A__quickstats.nass.usda.gov_&d=DwMGaQ&c=nE__W8dFE-shTxStwXtp0A&r=5AXco7ZLqTsBS70xUjh8U7kVmmSuIZA6S99nBEpjQjo&m=ZNPR9Rhq3AEs7151S_13tuGgl72byvNaJT-MRhBCpms&s=ZULIUr_g-6hz35e0EgzCEk9gMxlmPCRwWMtIPc69RlQ&e=))

^b^ Loss due to aflatoxin is calculated as the product of aflatoxin-related indemnities per hectare, planted corn area, and markup. Markup adjusts actual indemnity claims to estimate the underlying loss given that crop insurance contracts stipulate deductibles of about 30% of expected yield. We use the markup range 1.43 to 2.

Supplementary Table 7. Two years average Bt adoption rate by crop reporting district (%).

See Supplementary_Table.xlsx

Supplementary Table 8. Summary statistics and data sources.

| Variable | Definition | Mean | St. Dev. | Data source | Availability | Unit |
| --- | --- | --- | --- | --- | --- | --- |
| Aflatoxin occurrence rate | Aflatoxin claimed area over insured area*100 | 0.26^a^ | 2.51 | USDA RMA | 2001-2016 | County |
| Bt adoption | Bt/Stacked gene adoption rate | 52.5 | 30.7 | GfK | 2001-2016 | Crop-District |
| seed cost per expected yield | Ratio of average seed cost per unit to average yield between 1991-2000 ($ per bushels) | 0.59 | 0.28 | GfK & NASS | 2001-2016 | Crop-District |
| Expected yield | Average yield in each crop district in 1991-2000 (bu/hectare) | 280 | 62.1 | NASS | 1991-2000 |  |
| Palmer Z INDX (JUN) | Palmer Z index in June | 0.20 | 2.28 | NOAA | 1895-2016 | Climate District |
| Palmer Z INDX (JUL) | Palmer Z index in July | 0.17 | 2.28 | NOAA | 1895-2016 | Climate District |
| Palmer Z INDX (AUG) | Palmer Z index in August | 0.30 | 2.19 | NOAA | 1895-2016 | Climate District |
| Palmer Z INDX (SEP) | Palmer Z index in September | 0.11 | 2.13 | NOAA | 1895-2016 | Climate District |
| Favorable temperature (JUNE) | Ratio of the number of days with maximum temperatures between 30-40°C to the number of measured days | 0.55 | 0.26 | NOAA | 1763-2016 | Weather station |
| Unfavorable temperature (JUNE) | Ratio of the number of days with maximum temperatures above 42°C to the number of measured days | 0.00 | 0.01 | NOAA | 1763-2016 | Weather station |
| Favorable temperature (JULY) | Ratio of the number of days with maximum temperatures between 30-40°C to the number of measured days | 0.70 | 0.25 | NOAA | 1763-2016 | Weather station |
| Unfavorable temperature (JULY) | Ratio of the number of days with maximum temperatures above 42°C to the number of measured days | 0.00 | 0.01 | NOAA | 1763-2016 | Weather station |
| Aflatoxin related indemnities | Indemnity amount caused by aflatoxin ($1000) | 10.7 | 130 | USDA RMA | 2001-2016 | County |
| Insurance coverage | Weighted average of insurance coverage | 0.68 | 0.07 | USDA RMA | 1980-2016 | County |

^a^ This is the simple mean of aflatoxin occurrence rate. The weighted average is 0.13 (%) for 12,127 observations.

Supplementary Table 9. First stage of main regression: Instrumental variables on Bt adoption.

See Supplementary_Table.xlsx
